# A scoping review of “nursing-technology collaboration” in home-based rehabilitation for the elderly under the long-term care insurance system

**DOI:** 10.3389/fmed.2026.1906465

**Published:** 2026-09-25

**Authors:** Jingjing Sun, Weili Sun, Rui Xu, Linghui Zhang, Panpan Huai, Ruihua Jin, Huimei Lv

**Affiliations:** 1 School of Nursing, Shanxi Medical University, Taiyuan, China; 2 Shanxi Bethune Hospital, Shanxi Academy of Medical Sciences, The Third Hospital of Shanxi Medical University, Tongji Shanxi Hospital, Taiyuan, China; 3 Department of Rehabilitation Medicine, Shanxi Provincial People’s Hospital, Taiyuan, Shanxi Province, China

**Keywords:** elderly, home-based rehabilitation, long-term care insurance system, nurse-technology collaboration, scoping review

## Abstract

**Background:**

Under the long-term care insurance system, home-based rehabilitation is a key service model for addressing population aging, but it faces the dual challenges of a shortage of care workers and increasingly complex service needs. The introduction of emerging tools, such as digital health technologies and social assistive robots, has opened up new possibilities for home-based rehabilitation. However, whether technology can truly improve care outcomes depends on the collaborative working model between nurses and technology—known as “nurse-technology collaboration.” There is currently a lack of a systematic review of collaborative models in this field.

**Objective:**

To systematically analyze the phased positioning, nursing value, and institutional barriers to nursing-technology collaboration in home-based rehabilitation for the elderly under the long-term care insurance system, using a three-stage framework (tool substitution—capacity enhancement—ecological integration) based on the digital transformation maturity model and the theory of innovation diffusion, thereby providing a theoretical basis for technological integration and institutional innovation.

**Methods:**

Following the Joanna Briggs Institute's guidelines for systematic review methodology, we searched the PubMed, Cochrane Library, Web of Science, ScienceDirect, CINAHL, and Embase databases, with a search cutoff date of April 2026. We included studies that addressed the collaborative application of nursing staff and technology in home-based rehabilitation for the elderly, exploring collaborative models or mechanisms. We used descriptive analysis to synthesize the extracted data.

**Results:**

A total of 15 studies from 10 countries were included. In terms of phasing, five papers are in the tool replacement phase, ten are in the capability enhancement phase, and the ecosystem integration phase requires further exploration. In terms of application scenarios, nursing-technology collaboration has demonstrated measurable effectiveness across three core areas: physiological monitoring, risk warning, and rehabilitation training. Among these, evidence for physiological monitoring and risk warning is relatively concentrated, while research on motion quality supervision in rehabilitation training still has considerable room for improvement. In addition, other scenarios, such as social companionship and cognitive training, have positive implications for the mental health and social connections of older adults. In terms of key breakthroughs, data sharing and system interoperability, evaluation criteria and payment methods, digital literacy and talent development, and the delineation of responsibilities and institutional incentives are the four key issues that require focused attention.

**Conclusion:**

Currently, the collaboration between nursing and technology is primarily focused on tool replacement and capability enhancement, and has not yet achieved ecological integration. In the future, we should conduct practical randomized controlled trials based on performance-based payment models, develop technologies for monitoring the quality of nursing procedures, promote the standardization of data interfaces and training in digital literacy for nursing staff, and clarify accountability for technology use, thereby advancing the collaboration between nursing and technology toward a phase of ecological integration.

**Systematic Review Registration:**

https://doi.org/10.17605/OSF.IO/9RGWJ

## Introduction

The global population structure is undergoing unprecedented demographic changes, and population aging has become a widespread phenomenon. According to United Nations projections, by 2050, the global population aged 60 and over will exceed 2 billion, accounting for 16.4% of the total population (1). As people age, issues such as an increased prevalence of chronic diseases, physical decline, and cognitive decline become increasingly prominent (2). This has led to an increasing number of older adults needing to rely on others for daily care. Home-based rehabilitation has become a priority service model in many countries due to its ability to meet older adults' preference for aging in place, reduce the costs of institutional care, and maintain family and social connections (3, 4).

The rapid development of emerging technologies, such as digital health, artificial intelligence, the Internet of Things, and social assistive robots, has brought new solutions to home-based rehabilitation. From remote monitoring devices and smart pill boxes to social and telepresence robots, various tools are gradually entering the homes of older adults, playing important roles in medication reminders, fall detection, rehabilitation training, and social companionship (5–8). A large body of research has shown that whether technology can truly improve the outcomes of home-based rehabilitation depends on the collaborative working model between technology and nursing staff, i.e., “nursing-technology collaboration” (9). “Nursing-technology collaboration” refers to the formation of clear task division, information sharing, joint decision-making, and emergency response mechanisms between nursing staff (including professional nurses, therapists, family caregivers, etc.) and technological systems (such as robots, sensors, digital platforms), thereby achieving an organic integration of technology and personnel-based care (10, 11). From the perspective of nursing practice, the collaboration between nursing and technology combines the precision of technology with the professional judgment of nursing staff, overcoming the limitations of relying solely on technology or manual care. This approach enhances rehabilitation outcomes, ensures patient safety, and optimizes the allocation of nursing resources (7).

However, one study noted that the implementation and adoption of digital nursing technologies face challenges such as insufficient training, increased workload, and low technology confidence, indicating that, for nursing-technology collaboration to truly realize its value, a clear understanding of the different stages and characteristics of technology integration is required (12). The stepwise evolution of new technologies from initial trial to deep internalization, as outlined by the Diffusion of Innovations Theory, provides a robust theoretical framework for systematically analyzing the developmental stages of nursing-technology collaboration (13). Drawing on this theory and considering the characteristics of nursing practice, the developmental levels of nursing-technology collaboration can be divided into several stages, from low to high. These stages generally follow an evolutionary logic in which technology progresses from replacing manual labor in simple tasks to being embedded in workflows to assist decision-making, and finally to automatically interfacing with external institutional processes. In the field of elderly care, scholars have established a stage model of information technology maturity for nursing homes, providing a systematic tool for assessing the progressive levels of technology integration (14). Therefore, it is necessary to systematically identify the specific stages of nursing-technology collaboration in home-based rehabilitation for the elderly, the roles and competencies required of nursing staff at different stages, and the nursing scenarios applicable to each stage, in order to provide guidance for nursing staff to effectively use technology and facilitate their professional role transformation.

The practice of nursing-technology collaboration inevitably involves issues of cost and payment, as well as resource allocation, during technology implementation and sustained service delivery. Fragmented payment mechanisms, fluctuating regulatory policies, and the lack of incorporation of nursing services into an independent reimbursement system have become key bottlenecks to large-scale promotion (15). From an economic perspective, the long-term care insurance system, through social insurance or tax-financed approaches, provides financial support and service coverage for home-based, community-based, and institutional care services for disabled and semi-disabled older adults, thereby serving as a key measure to facilitate the integration of technology into home-based rehabilitation (16). The target population covered by long-term care insurance closely aligns with the service recipients of home-based rehabilitation; both primarily serve individuals whose ability to perform activities of daily living has declined due to aging, illness, or disability, and who consequently require long-term care support. Currently, the reimbursement criteria for long-term care insurance are largely based on service frequency or duration, lack valid measures of service quality and outcomes, and fail to adequately reflect cost variations across different levels of nursing-technology collaboration. In terms of international trends, member countries of the Organization for Economic Co-operation and Development are gradually moving toward a hybrid model that combines fee-for-service with value-based payment linked to health outcomes (17). In China, the National Healthcare Security Administration (NHSA) has issued a unified catalog of long-term care insurance service items, yet technology-assisted services—such as smart rehabilitation and remote monitoring—have not been fully incorporated into the reimbursement system. The system is currently undergoing a critical transition from pilot programs to nationwide institutional implementation (18, 19). Objective data generated during nursing-technology collaboration, such as continuous physiological monitoring indicators, risk alert success rates, and adherence to rehabilitation regimens, can provide a scientific basis for shifting long-term care insurance from per-service payment to outcome-based payment. Therefore, this scoping review will systematically analyze the stage positioning, application scenarios, and key breakthroughs of nursing-technology collaboration in home-based rehabilitation for the elderly under the long-term care insurance system, in order to provide a theoretical basis for technology integration and institutional innovation, and to promote the synergistic optimization of nursing practice and the insurance system.

## 2 Methods

### 2.1 Study design

This scoping review followed the Joanna Briggs Institute methodology for scoping reviews (20). This scoping review was reported in accordance with the Preferred Reporting Items for Systematic Reviews and Meta-Analyses extension for Scoping Reviews (PRISMA-ScR) checklist (21). The scoping review protocol was pre-registered on the Open Science Framework (OSF) at https://osf.io/9rgwj. This methodology has been widely used in nursing research to explore and analyze complex issues, as it facilitates comprehensive mapping of the literature and supports the development of conceptual frameworks (22). By adopting this approach, the scoping review ensures a systematic and transparent process.

### 2.2 Research process

#### 2.2.1 Defining the research question

This scoping review focuses on three main research questions: (a) **Stage positioning:** At which stage (tool substitution, capability enhancement, or ecological integration) does nursing-technology collaboration in home-based rehabilitation under the current long-term care insurance system mainly reside?; (b) **Application scenarios and nursing value:** In which specific nursing scenarios of home-based rehabilitation has nursing-technology collaboration been applied? How does it bring practical value to nursing work? What areas for improvement currently exist in each scenario?; (c) **Directions for optimization and key breakthroughs:** Based on existing research, what critical bottlenecks need to be overcome for the further development of nursing-technology collaboration? What effective countermeasures can be taken to address these bottlenecks?

#### 2.2.2 Identification of relevant research

A comprehensive search strategy was developed and applied to six major databases from inception to April 2026: PubMed, Cochrane Library, Web of Science, ScienceDirect, CINAHL, and Embase. The complete search queries for each database are provided in Supplementary File 1. The search strategy combined free-text terms and Medical Subject Headings (MeSH), with Boolean operators used to ensure comprehensive retrieval of relevant literature. In addition, grey literature was identified through Google Scholar, and both backward and forward citation chasing were performed for all included studies.

To establish the inclusion and exclusion criteria, the Population–Concept–Context (PCC) framework was adopted (20). The Population refers to community-dwelling older adults under the long-term care insurance system, including those who are disabled or semi-disabled. The Concept is the “nursing-technology collaboration” relationship, i.e., the collaborative model between nursing staff (professional nurses, therapists, family caregivers, etc.) and technological systems, encompassing human-machine collaboration and technology-assisted nursing. The Context includes the institutional background (the long-term care insurance system) and service settings (home-based rehabilitation, home-based nursing care, community-based rehabilitation, etc.).

Inclusion criteria were as follows: (a) Articles involving the collaborative application of nursing staff and technology in home-based rehabilitation for the elderly; (b) Articles addressing the “nursing-technology collaboration” model, task division, collaboration mechanisms, or influencing factors; c) Articles including but not limited to qualitative studies, quantitative studies, mixed-methods studies, and systematic reviews.

It should be noted that although some of the included studies were conducted in nursing homes or long-term care facilities, their intervention models, technological applications, and care coordination mechanisms (such as remote monitoring, medication management, and social companionship) are clearly applicable to home-based rehabilitation, and the study findings can be directly generalized to home settings. In data extraction, this review focused on intervention content related to home-based rehabilitation and elaborated on the generalizability of such studies in the discussion section.

Exclusion criteria were as follows: (a) Abstracts and conference papers; (b) Articles that mention the topic only in the title, abstract, or keywords without substantive discussion in the main text; (c) Articles for which the full text is not available; (d) Articles that focus solely on technology development or robotic hardware design without discussing the collaborative application of nursing staff and technology; (e) Articles whose study population is not older adults (age < 65 years) or whose setting is not home-based rehabilitation (e.g., entirely in hospitals or nursing homes without any home-based element); (f) Articles that only examine older adults' acceptance of technology or the effectiveness of technology, without addressing the role of nursing staff or collaboration mechanisms.

### 2.3 Study selection

All identified records were collated and uploaded into EndNote software to remove duplicate records. This process was performed independently and blinded by two researchers (Sun Jingjing and Xu Rui). Literature screening was strictly conducted according to the inclusion and exclusion criteria, and reasons for exclusion were documented during the full-text screening phase. Given the multidisciplinary nature of this study, the researchers standardized the screening criteria. They reached a consensus on interdisciplinary terms related to “nursing-technology collaboration” (e.g., nursing staff–technology collaboration, human-machine collaboration, remote monitoring, social assistive robots, etc.) to ensure screening reliability during the title/abstract screening stage. Any disagreements arising at each stage of the study selection process were resolved through team discussion until consensus was reached.

### 2.4 Data extraction and data analysis

This scoping review followed the JBI data extraction and analysis approach. A data extraction tool (i.e., Microsoft Word) was used to collect information from the included studies, including author, publication year, country of publication, document type, research objective, study population, nursing-technology collaboration technologies/tools involved, core collaboration models, targeted collaboration outcomes, application scenarios, main findings/conclusions, and limitations. This process was performed by two independent researchers, and a third researcher cross-verified the extracted data to reduce bias. For data analysis, a descriptive approach consistent with the review objectives was adopted. This approach enables a comprehensive analysis that integrates logical and descriptive components, aligns with the review objectives, and facilitates the identification of gaps in the literature (23).

## 3 Results

The initial search yielded 146 articles. After removal of duplicates, 125 articles remained. Subsequently, 31 articles were excluded after title and abstract screening, and a further 79 articles were excluded after full-text review, resulting in the inclusion of 15 articles in this review. Figure 1 presents the PRISMA flow diagram detailing the study selection process.

**Figure 1:**
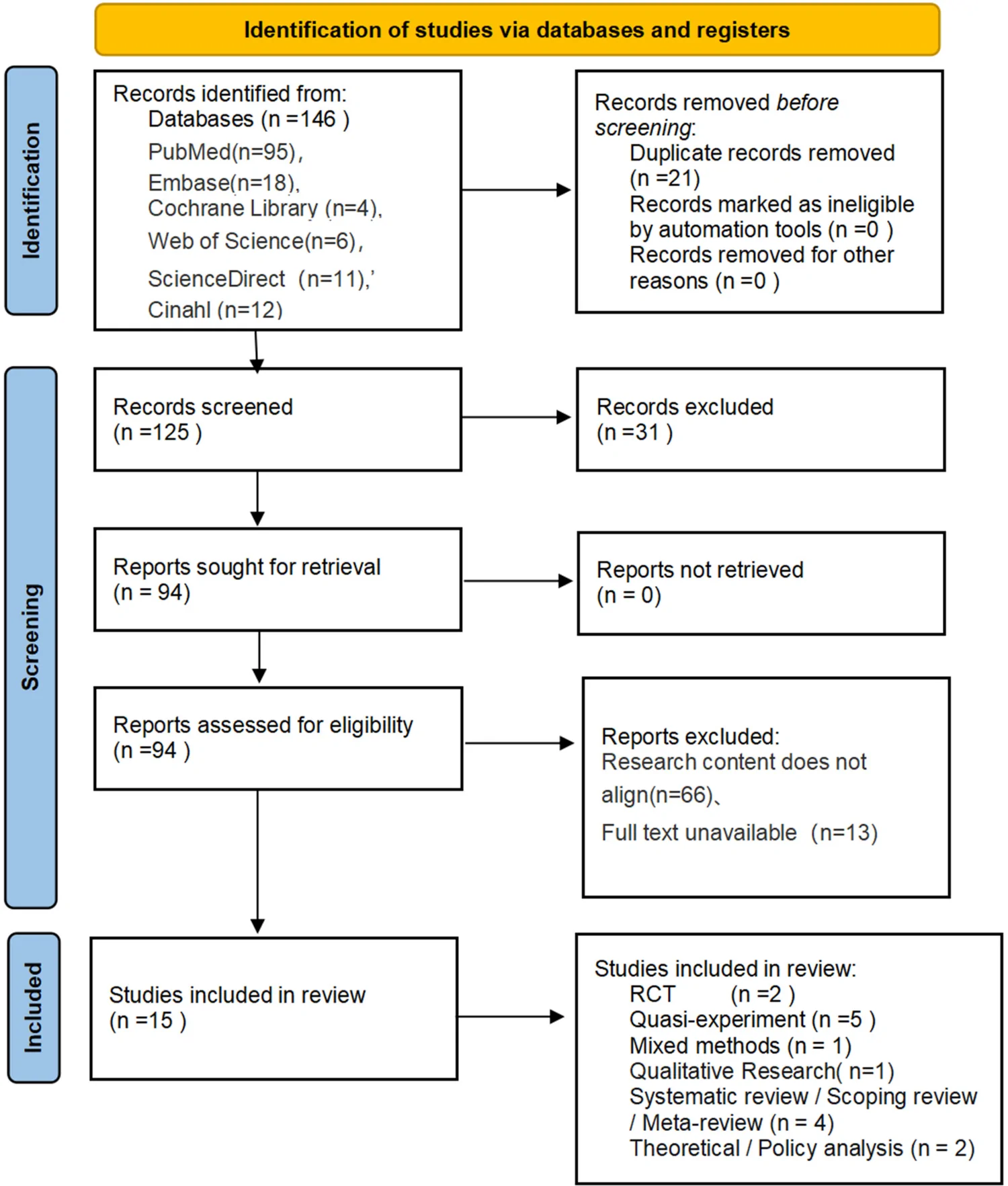
PRISMA flowchart.

### 3.1 Study characteristics

Among the 15 included articles (24–38), all were published between 2016 and 2026. The included studies originated from ten countries: South Korea (*n* = 2) (24, 25), China (*n* = 2) (26, 27), Australia (*n* = 1) (28), Japan (*n* = 1) [ (29), Spain (*n* = 1) (30), Germany (*n* = 1) (31), Italy (*n* = 2) (32, 33), Canada (*n* = 2) (34, 35), North Macedonia (*n* = 2) (36, 37), and Finland (*n* = 1) (38). Study types included randomized controlled trials (2 studies), quasi-experimental studies (5 studies), mixed-methods studies (1 study), qualitative studies (1 study), systematic reviews/scoping reviews (4 studies), and theoretical/policy analyses (2 studies). Detailed characteristics of the included studies are presented in Table 1.

**Table 1:** Basic information on the included studies (*n* = 15).

No.	Authors	Year	Country	Document type	Study population	Nursing-tech collaboration technologies/tools	Stage positioning	Application scenarios (monitoring/alert/rehabilitation)	Key breakthroughs
1	Yoo Keunjoo (24)	2022	South Korea	Secondary data analysis	131 community-dwelling older adults	ICT-enhanced home visiting nursing	Capability enhancement	Monitoring: continuous data collection + remote physician decision-making	Data sharing and system interoperability
2	Ivy Yan Zhao (26)	2025	Hong Kong, China	Mixed-methods study	30 community-dwelling older adults	Social robot KaKa	Tool substitution	Rehabilitation: medication reminders, exercise guidance; Monitoring: companionship data recording	Data sharing and system interoperability; Digital literacy and workforce development
3	Elizabeth (28)	2018	Australia	Randomized controlled trial	60 COPD patients post-discharge	Social assistive robot iRobi + remote monitoring	Capability enhancement	Monitoring: COPD physiological indicators; Alert: symptom deterioration; Rehabilitation: video guidance + adherence improvement	Evaluation criteria and payment models
4	Zeng Yixuan (27)	2025	China	Integrative review	11 articles	Digital platforms, information flow management tools, workflow collaboration systems	Capability enhancement	Monitoring: multi-stakeholder information sharing	Data sharing and system interoperability
5	Han Wool Jung (25)	2026	South Korea	Prospective single-arm intervention study	278 community-dwelling older adults	Hyodol social assistive robot	Capability enhancement	Monitoring: daily health checks; Alert: abnormal triggers intervention; Rehabilitation: behavioral activation	Data sharing and system interoperability
6	Keita Kiuchi (29)	2023	Japan	Systematic meta-review	315 systematic reviews	Conversational agents, chatbots, SARs	Capability enhancement	Monitoring/Rehabilitation: emotional companionship, cognitive training	Responsibility allocation and institutional incentives
7	Belen Liedo (30)	2025	Spain	Ethical analysis	ROB-IN assistive robot (Spanish publicly funded project, intended users: non-disabled older adults receiving public home assistance)	ROB-IN assistive robot	Capability enhancement	Monitoring: information collection and sharing	Responsibility allocation and institutional incentives
8	Marc (31)	2025	Germany	Qualitative interview study (constructivist grounded theory)	12 nursing staff + 4 stakeholders	Social assistive robots (including Lio, Navel, Paro, Pepper, JAIme, YANNY, etc.)	Capability enhancement	Rehabilitation: social facilitation, memory training	Digital literacy and workforce development; Data sharing and system interoperability
9	Milella (32)	2024	Italy	Systematic review	24 articles	AI ambient assisted living, sensor networks, robots	Capability enhancement	Monitoring: remote monitoring; Coordination: formal and informal caregivers	Evaluation criteria and payment models
10	Macdonald (34)	2024	Canada	Mixed-methods systematic review	Residents of long-term care facilities	Videoconferencing, tablets, digital storybooks	Tool substitution	Rehabilitation: social connection (assisted by nursing staff); Other: social companionship	Digital literacy and workforce development
11	Koceska (36)	2019	North Macedonia	Technology development and validation study (simulated and real-world experiments)	26 older adults + 5 professional nurses	Low-cost telepresence robot	Tool substitution	Alert: remote observation; Rehabilitation: grasping assistance; Other: social facilitation	Data sharing and system interoperability
12	Saso Koceski (37)	2016	North Macedonia	Technology validation and user acceptance study	30 older adults + 5 nursing staff	Telepresence robot	Tool substitution	Monitoring: ECG measurement; Rehabilitation: grasping assistance	Data sharing and system interoperability; Digital literacy and workforce development
13	Lorie Donelle (35)	2025	Canada	Pragmatic randomized controlled trial	313 home care client–caregiver dyads	Passive remote monitoring system	Capability enhancement	Monitoring: activity/medication/falls; Alert: real-time alert + response closed loop	Responsibility allocation and institutional incentives
14	Mariano Cingolani (33)	2023	Italy	Policy/opinion review	Italian home care services	AI decision support systems, remote monitoring, robots, etc.	Capability enhancement	Monitoring/Rehabilitation: multi-stakeholder collaboration	Data sharing and system interoperability; Responsibility allocation and institutional incentives
15	Rantanen (38)	2017	Finland	Prospective safety and usability trial	17 nursing home patients + 27 home-dwelling patients	Evondos E300 medication dispensing robot	Tool substitution	Rehabilitation: medication adherence (98.7% on-time pill retrieval)	Data sharing and system interoperability; Evaluation criteria and payment models

### 3.2 Stages of digitalization in “nursing-technology collaboration”

Drawing on the hierarchical logic of the digital transformation maturity model (GB/T 39116-2020) (39) and the staged evolution perspective of the Diffusion of Innovations Theory (13), which describes the process by which new technologies gradually move from initial trial to deep internalization and ecological integration, and taking into account the characteristics of nursing practice, this study classifies the developmental levels of “nursing-technology collaboration” into three progressive stages from low to high: ①Tool substitution stage: At this stage, technology is used only to replace manual labor in performing simple, repetitive single tasks, such as medication reminders, one-button calls, GPS positioning, and environmental monitoring. Five studies (26, 34, 36–38) fell into this stage, in which technology only substituted for manual labor in repetitive single tasks, without being embedded in a nursing decision-making closed loop. For example, the telepresence robots in Koceska (36)and Koceski (37)were remotely controlled by nursing staff to grasp objects, and the navigation function was difficult for older adults to operate. The social robot in Zhao (26)provided reminders and companionship, but the data were only for manual review. The medication dispensing robot in Pekka (38)achieved a 98.7% on-time pill retrieval rate, yet the response protocol was not standardized. Macdonald (34)found that older adults required substantial human assistance when using video calling. ②Capability enhancement stage: At this stage, technology is gradually embedded into nursing workflows and can automatically collect health data (e.g., heart rate, activity level, sleep quality), providing supportive assistance for nursing staff's professional judgment and care decisions. Ten studies (24, 25, 27–33, 35)involved this stage, in which technology was embedded into processes to form a bidirectional, closed loop and assist decision-making. For example, in Yoo et al. (24) In ICT-enhanced home visiting nursing, remote conferencing achieved better outcomes than mere data sharing. The iRobi robot in Elizabeth (28) improved medication adherence from 29.5% to 48.5%. The PRM system in Donelle (35)reduced emergency department visits by 65%. The Hyodol robot in Jung et al. (25)the risk of high depression by 45%. Theoretical reviews by Zeng Yixuan (27), Milella (32), and Mariano (33)described the concept of platform-based collaboration. Three other theoretical articles were also included: Kiuchi (29)conducted a systematic meta-review summarizing ethical and practical challenges of social assistive robots in elderly care; Liedo (30)provided an ethical analysis pointing to issues of robot autonomy, privacy, and responsibility boundaries; and Marc (31)proposed a triadic care model through a qualitative study, clarifying that robots should not be involved in emotional support or end-of-life care. Although these three articles did not provide quantitative effect data, they offered theoretical support for the collaboration mechanisms at the capability enhancement stage. ③Ecological integration stage: At this stage, technology data are used not only to support decision-making in personnel-based care but also to automatically interface with institutional processes such as payment, regulation, and service adjustment within the long-term care insurance system. Examples include medication records being automatically used for service-volume reimbursement, and health-improvement data serving as the basis for outcome-based payment. Among the included studies, no empirical case has been reported in which technology data were automatically interfaced with the payment and regulatory systems of long-term care insurance. Only Zeng Yixuan (27)and Cingolani (33)mentioned in their theoretical analyses the possibility of such automatic interfacing (e.g., medication records directly applied to cost settlement, health-improvement data used for outcome-based payment), but neither was implemented in real-world cases.

### 3.3 Application scenarios and nursing value of “nursing-technology collaboration”

Based on the core elements of nursing practice (physiological status monitoring, safety risk prevention, and rehabilitation function promotion) and drawing on the content of the included studies, three main application scenarios were identified. In addition, some studies addressed areas such as social companionship and cognitive training, which were categorized as other scenarios. ①Physiological monitoring: Six studies (24, 25, 28, 35–37) utilized wearable devices or environmental sensors to achieve 24-hour continuous collection of data such as heart rate, activity level, and sleep. Yoo et al. (24), Donelle et al. (35), and Jung et al. (25) employed wearable devices or environmental sensors to collect heart rate, activity level, and sleep data. However, a limitation is that the data are used only for manual review or simple alerts and have not been linked to automatic adjustments in the frequency of long-term care insurance services. ②Risk warning: Three studies (28, 30, 35)utilized technologies such as fall alerts and COPD symptom monitoring to significantly reduce emergency response time. Donelle et al. (35) reported that fall alerts decreased emergency department visits by 65%; Broadbent et al. (28) achieved early intervention through COPD symptom warnings. However, once a warning is triggered, the response still relies on manual judgment by caregivers, leading to blurred responsibility boundaries. In Donelle et al.'s (35)study in Nova Scotia, due to distorted institutional incentives (over-response to gain priority admission to facilities), the warnings paradoxically led to increased hospitalizations. Liedo (30) pointed out that the responsibility for missed warnings remains unclear. ③Rehabilitation training: Five studies (25, 26, 28, 31, 38) improved patient adherence and engagement through video guidance, medication reminders, and social companionship. Broadbent et al. (28) reported that video guidance increased exercise frequency; Rantanen et al. (38) found that medication reminders achieved a 98.7% on-time pill retrieval rate; Zhao et al. (26) showed that robotic exercise guidance enhanced participation. A limitation is that the technology primarily addresses whether the patient performs the activity, but cannot assess how well it is performed, lacking biosignal-based motion quality monitoring. Bubeck et al. (31) pointed out that emotional support and complex judgments during rehabilitation still rely on nursing staff. ④Other scenarios [2 studies (34, 36)]: These involve areas such as social companionship, cognitive training, and emotional support. The review by Kiuchi et al. (34) indicated that social assistive robots can provide emotional companionship and cognitive stimulation. The study by Bubeck et al. (36) emphasized that robots can be used for social facilitation and memory training, but emotional support and end-of-life care still need to be delivered by nursing staff. Although these scenarios are not directly related to rehabilitative care, they have positive implications for the mental health and social connection of older adults.

### 3.4 Key breakthroughs of “nursing-technology collaboration”

From the 15 included studies, the main aspects affecting the further development of nursing-technology collaboration were summarized into the following four breakthrough areas: ① Data sharing and system interoperability: Five studies (27, 32–34, 38) pointed out that device data formats are inconsistent and do not interoperable with insurance systems. Cingolani (33) noted that the adoption of electronic health records in Italy is slow, and information silos are severe. Zeng Yixuan (27) emphasized the difficulty of cross-institutional data sharing. Rantanen (38) observed that medication records and Donelle (35) found that activity trajectories could not be automatically imported into reimbursement systems. ②Evaluation criteria and payment models: Four studies (27, 28, 32, 33) pointed out the lack of evaluation criteria for rehabilitation outcomes with technology assistance and the absence of outcome-based payment mechanisms. Milella (32) noted the lack of unified evaluation indicators. Broadbent et al. (28) only mentioned cost-effectiveness analysis without developing an operable model. Cingolani (33) emphasized that the unclear attribution of responsibility for AI-based decisions hinders the transition in payment systems. ③Digital literacy and workforce development: Five studies (26, 31, 34, 35) pointed out the insufficient digital literacy of both nursing staff and older adults. Macdonald (34) found that older adults required substantial assistance to use devices. Zhao (26) and Koceski (37) had sample bias (high educational level, prior technical experience), which underestimated the real barriers. Bubeck (31) noted that nursing staff faced increased burden due to technical failures and lacked training in data interpretation. ④Responsibility allocation and institutional incentives: Four studies (30, 31, 33, 35) pointed out that the responsibility for missed warnings is unclear, and institutional incentives may lead to distortions. Donelle (35) found that in Nova Scotia, caregivers over-responded to gain priority admission to facilities, resulting in negative outcomes. Liedo (30) emphasized the risks to privacy and to paternalism. Cingolani (33) proposed that legal clarification is needed regarding the responsibility pathways for AI-assisted decision-making.

## 4 Discussion

### 4.1 The evolution trajectory of “nursing-technology collaboration” and the reshaping of the nurse’s role in the digital age

Based on an integrated framework combining the digital transformation maturity model and the Diffusion of Innovations Theory, this scoping review found that nursing-technology collaboration practices are mainly situated at the tool substitution and capability enhancement stages, whereas the ecological integration stage, which represents a higher level of integration, was not covered in the included literature (40). The relative slowness of this evolutionary process may be related to the maturity of cross-institutional governance mechanisms and payment-side support policies, such as long-term care insurance (16).

The realization of technological efficacy depends, to a certain extent, on the specific application scenario. In the tool substitution stage, technology primarily alleviates the shortage of frontline nursing staff by automating standardized tasks (e.g., medication reminder devices, emergency call systems). Such technology can meet the daily care needs of older adults with mild disabilities and reduce the care burden on family members, but its capacity to improve functional recovery is limited (38, 41). In the capability enhancement stage, technology leverages dynamic assessment and decision-support functions (e.g., chronic obstructive pulmonary disease management, fall risk alerts) (29, 33, 35) to drive a shift in the nursing model from passive response to active intervention (36). However, the effective functioning of capability enhancement technologies is not automatic; it depends on nursing staff possessing strong data interpretation skills and clinical judgment. If nurses merely view the data mechanically without understanding the underlying pathophysiological significance, no amount of monitoring data can be translated into effective nursing interventions. Therefore, this stage places substantial demands on nurses' digital literacy.

This study further clarified the task division under the nursing-technology collaboration model. The core responsibilities of nurses primarily include on-site assessment and nursing procedures, remote monitoring and active intervention, issuing high-level instructions, responding to alerts, conducting home visits, medication management, and human-robot integration and emotional support in social robot assistance. Technology, on the other hand, is mainly responsible for standardized tasks such as data collection and recording, information transmission, low-level automated execution, and alert transmission (20, 24–28, 42). These divisions indicate that regardless of how technology evolves, nurses consistently undertake core functions such as on-site assessment, emergency decision-making, human-robot coordination, and emotional support, while technology is responsible for standardizable tasks including data collection, information transmission, low-level automation, and alert delivery. Therefore, the essence of nursing-technology collaboration is not technology replacing nurses, but rather achieving complementary advantages through a clear division of labor. However, the nursing role's positioning may exhibit stratified characteristics depending on the institution's level and the service recipients. In primary community health service centers, nurses are more often tasked with device operation, anomaly identification, and basic rehabilitation guidance. In secondary or higher-level rehabilitation institutions or professional home-based nursing teams, nurses need to progressively develop into data-driven clinical decision-makers, possessing capabilities in trend analysis, remote intervention, and interdisciplinary coordination (31). This role stratification imposes differentiated demands on nursing education, job training, and career development pathways (43, 44).

In addition, attention should be paid to the potential risk of deprofessionalization associated with the application. At the tool substitution stage, nursing work may be externally misperceived as low-skilled repetitive labor. At the capability enhancement stage, some nursing outcomes may be attributed to algorithmic or device functions rather than to nurses' clinical judgment and humanistic care (30). Such a deviation in value perception may adversely affect nursing professional identity and may also lead to biases in resource allocation (41). Therefore, it is particularly important to strengthen the recognition of the core values of the nursing profession at the institutional level. While ensuring technology empowerment, the humanistic care and clinical judgment abilities of nursing also need to be valued (45).

With the gradual implementation of the long-term care insurance system, community nurses are expected to become key actors bridging technology and nursing management (16). Future efforts should not be limited to technological development alone. Instead, systematic institutional design should be employed to enhance the digital literacy and cross-system coordination capabilities of primary care nurses, clarify the rules for allocating responsibility for technology use, and reduce underutilization or inefficient use of technology in grassroots practice (43, 44). Only then can nursing-technology collaboration achieve an orderly progression from tool substitution to capability enhancement and ultimately to ecological integration, thereby ensuring the continuation and enhancement of nursing values in the digitalization process.

### 4.2 The nursing value of “nursing-technology collaboration” in comprehensive nursing scenarios

This scoping review found that collaboration between technology and nursing staff delivers measurable value across three core nursing scenarios. In addition, related areas such as social companionship and cognitive training also have positive implications for the mental health and social connection of older adults, representing other scenarios of particular interest in this review.

In the physiological monitoring scenario, continuous data collection enables nurses to shift from traditional intermittent home visits to real-time remote monitoring, facilitating earlier detection of health abnormalities and corresponding adjustments to care plans (24, 25, 28, 35–37). This shift not only enhances the timeliness of nursing care but also generates objective functional maintenance indicators (e.g., stable heart rate, achieved activity levels, etc.), which can serve as a potential basis for outcome-based payment in long-term care insurance (46). Secondly, long-term care insurance could incorporate the rate of achieving physiological control targets into the performance assessment of community nursing institutions, thereby incentivizing nurses to focus on routine health management rather than merely completing the number of home visits (47). However, the linkage mechanism between monitoring data and the frequency of insurance services has not yet been established, and the efficiency of translating data into nursing actions remains suboptimal.

In the risk warning scenario, early warning systems enable nurses to receive alerts before or during emergency events, thereby shortening emergency response time and enhancing safety for older adults living alone (28, 30, 35). The success rate of technology alerts and the timeliness of nurse responses can serve as indicators of quality for long-term care insurance. For example, completing telephone guidance within 30 min of a fall alert could be set as one of the payment conditions, thereby incentivizing community nursing institutions to establish rapid-response mechanisms. Meanwhile, the cost savings from emergency hospitalizations avoided by alerts could be partially returned to nursing institutions. Currently, after an alert is triggered, the response still largely relies on caregivers' manual judgment within the nursing workflow, lacking a standardized graded response protocol. This results in blurred responsibility attribution, leading to alert fatigue and delayed responses (48). In the future, it is necessary to more deeply integrate technology alerts with nursing workflows and optimize the graded response system.

In rehabilitation training, technology-assisted tools such as video guidance and biofeedback help improve patient adherence to rehabilitation regimens. Rehabilitation training entails differentiated needs across populations. Patients undergoing pulmonary rehabilitation require monitoring of respiratory rate, blood oxygen saturation, and expectoration ability; those in cardiac rehabilitation need to control their heart rate range and avoid overexertion; and older adults receiving cognitive rehabilitation need to engage in cognitive training involving memory, calculation, and attention (25, 26, 28, 31, 38). In terms of integration with long-term care insurance, the outcomes of rehabilitation training can be quantified through data such as training duration, task completion rates, and improvements in physiological indicators. Long-term care insurance could use the achievement rate of rehabilitation goals (e.g., increased 6-minute walking distance, improved cognitive scores) as a basis for adjusting payment levels, thereby promoting the transition from per-service payment to outcome-based payment.

In addition, positive intervention evidence has accumulated in areas such as social companionship and cognitive training. It is therefore necessary to consider these domains simultaneously within comprehensive nursing intervention protocols and progressively explore new pathways that integrate smart devices with professional nursing care (34, 36). In terms of nursing human resource allocation, the core of the long-term care insurance system lies at the home and community level, with its primary service recipients being disabled and semi-disabled older adults living in the community. Therefore, the main force undertaking daily home-based rehabilitation guidance tasks should be community nurses or nursing staff from primary health service centers. Hospital nurses are primarily responsible for pre-discharge assessment, formulation of rehabilitation plans, and acute-phase interventions. Community nurses, who have long-term contact with service recipients and are familiar with their living habits and health baselines, are better suited to utilize technological data for dynamic management and alert response (49). Therefore, the long-term care insurance payment system should prioritize supporting skill training, technical support, and performance incentives for community nursing staff, ensuring that nursing-technology collaboration can be effectively implemented at the grassroots level (50, 51).

On the other hand, when evaluating the effectiveness of the synergy between nursing and technology, the subjective experiences of older adults as technology users should not be overlooked. The literature currently included in this review primarily focuses on the synergistic relationship between nursing staff and technology, with little attention paid to older adults' acceptance of technology, their user experiences, and their satisfaction ratings. Older adults (regardless of whether they have functional impairments) are the direct beneficiaries of technology-based services, and their user experience and satisfaction should be one of the core dimensions in evaluating technology design and nursing care. A study by Curioso-Vílchez et al. (52) indicates that systematic assessments of assistive technology user satisfaction help identify the degree of alignment between technology and user needs, thereby guiding technology optimization and service improvements. At the same time, the design of home-based rehabilitation technologies should adhere to universal design principles to ensure that technological products and services can be used by older adults with varying functional statuses—including those who are disabled, partially disabled, or living with cognitive impairments—and to avoid creating new forms of digital exclusion due to complex interfaces, inconvenient operation, or high perceptual barriers (53, 54). Combining user experience feedback with evaluations of the synergistic effects between care and technology helps to more comprehensively reflect the actual value of the technology and provides a basis for subsequent technological iterations and improvements in nursing practice.

### 4.3 Influencing factors and coping strategies of “nursing-technology collaboration” under digital empowerment

This scoping review found that the development of nursing-technology collaboration is influenced by multiple factors, among which data sharing and system interoperability constitute the foundational link for bridging technology empowerment and institutional embedding. Currently, many rehabilitation devices use inconsistent data formats that cannot be automatically interfaced with the billing system of long-term care insurance, resulting in the underutilization of valuable monitoring data (27, 33). Inconsistent data standards across different devices and difficulties in achieving interoperability between institutions remain particularly prominent. Therefore, it has been suggested that unified data interface standards for home-based rehabilitation devices should be established, leveraging the unified national medical insurance information platform to enable automatic data upload and sharing (55). This would not only reduce the manual data entry burden on nursing staff but also provide a technical prerequisite for long-term care insurance to conduct payment reviews based on objective data.

The coordinated development of evaluation criteria and payment models is considered a key link in advancing nursing-technology collaboration toward the ecological integration stage. Chinese scholars' comparative studies of Germany, Japan, and South Korea have suggested that multidimensional assessment methods offer valuable insights for establishing unified assessment criteria in China (56). Meanwhile, the German long-term care insurance system prioritizes prevention and rehabilitation over bedridden nursing care, with home-based rehabilitation services assessed and delivered by professional teams through home visits (57). However, it is not realistic to directly copy international experiences; localized adaptation is needed based on the actual circumstances of pilot cities in China (58). Therefore, it is recommended to explore controlled trials of outcome-based payment on this basis, incorporating objective health indicators generated by nursing-technology collaboration, such as continuous physiological monitoring, functional maintenance, and risk reduction, into the determination of payment tiers, thereby promoting a gradual transition of long-term care insurance from per-service payment to payment based on health gains (59).

Digital literacy and workforce development are key supports for technology implementation. The majority of studies included in this review indicate that insufficient digital literacy among both nursing staff and older adults is an important factor affecting the effectiveness of technology application. For example, older adults require substantial assistance to use smart devices, and nursing staff, lacking training in data interpretation, find it difficult to effectively utilize monitoring results, and may even experience increased workload due to technical failures (26, 31, 34, 35). This indicates that simply procuring advanced equipment does not automatically improve the quality of care; human capital development must be strengthened concurrently. It is therefore recommended that the operation of smart nursing devices and data interpretation be incorporated into the continuing education system for nursing staff, and that efforts to establish a specialized digital nursing workforce draw on the experience of creating new professions such as health care workers and long-term care workers (60).

Clear responsibility allocation and institutional incentives are important safeguards for sound development. International experience suggests that the objective collection of continuous physiological monitoring, risk warning, and rehabilitation training data holds potential value for supporting dynamic disability grading and service package optimization (61, 62). However, the introduction of technology-generated data also raises new legal issues: when missed or false alerts lead to adverse incidents, should liability be borne by technology vendors, nursing institutions, or nurses? The relevant legal framework remains unclear at present. To address current challenges, it is necessary to clarify multi-tiered responsibility-allocation rules at the legislative and institutional design levels for cases of missed or false technology alerts, and to adjust relevant long-term care insurance policies in a timely manner to reduce the risk of potential system abuse (61, 62). China should accelerate the conduct of localized empirical studies on nursing-technology collaboration to validate the role of physiological monitoring data in disability grading assessment, the value of risk warning data for emergency care payment, and the feasibility of rehabilitation adherence data as a basis for outcome-based payment. Drawing on international experience, the long-term care insurance payment system should be gradually refined, thereby providing institutional and workforce support for the steady progression of nursing-technology collaboration from capability enhancement to ecological integration.

Future research could build upon the phased framework and key breakthroughs outlined in this review, adopting a combined analytical and argumentative approach to delve deeper into the subject. At the same time, it is necessary to examine the issue of collaboration between nursing and therapy in home-based rehabilitation from a holistic perspective, comprehensively considering diverse indicators such as the functional status, psychological needs, social environment, and technology acceptance of the elderly population, thereby advancing research in this field toward a more systematic and refined direction.

## 5 Limitations

This scoping review has several limitations. First, the number of included studies is small (only 15), and no empirical study was found at the ecological integration stage. Moreover, most studies at the capability-enhancement stage relied on manual intervention to close the loop, limiting the generalizability of conclusions about the advanced features of nursing-technology collaboration. Second, some of the included studies did not incorporate long-term care insurance system variables (e.g., payment methods, regulatory rules) as core analytical dimensions, weakening the evidence base for the moderating effects of institutional factors on collaboration outcomes. Third, while this review focuses on home-based rehabilitation, some studies were conducted in nursing homes or day care centers, where the physical environment and staffing differ from those in strictly home-based settings; thus, generalizing the findings requires caution. Fourth, the methodological quality of the included studies is variable, with only two randomized controlled trials, and most intervention periods are shorter than six months, lacking evaluations of long-term effects and sustainability of the collaboration models. Fifth, this review included only English-language literature. Although no language restrictions were set for the search, the studies ultimately included were all published in English, which may have resulted in the omission of relevant studies in other languages, such as Portuguese and Spanish, and thus introduced a certain degree of language bias. Sixth, the representativeness of the sample was limited; most participants had a high level of education and some technical experience. There is insufficient evidence regarding low-income older adults, those with low digital literacy, and rural older adults, which limits the generalizability of the conclusions to the entire older adult population.

## 6 Conclusions

The studies included in this scoping review indicate that nursing-technology collaboration in home-based rehabilitation for older adults under the long-term care insurance system has been implemented in several countries, including South Korea, Australia, Canada, and Italy. Currently, such collaboration is mainly situated at the tool substitution stage and the capability enhancement stage, while the ecological integration stage requires further exploration. In terms of application scenarios, nursing-technology collaboration has shown measurable effectiveness in three core scenarios: physiological monitoring, risk warning, and rehabilitation training. Among these, evidence for physiological monitoring and risk warning is relatively concentrated, whereas research on motion quality supervision in rehabilitation training still has considerable room for improvement. In addition, other scenarios such as social companionship and cognitive training also have positive implications for the mental health and social connection of older adults, serving as important complements to comprehensive home-based rehabilitation. Regarding key breakthroughs, data sharing and system interoperability, evaluation criteria and payment models, digital literacy and workforce development, and responsibility allocation and institutional incentives are four critical issues that need to be addressed.

In response to these challenges, future research could focus on the following aspects: strengthening theoretical guidance by embedding stage-specific progression goals based on the three-stage framework into intervention design; developing comprehensive evaluation tools that combine objective monitoring data with subjective indicators such as nursing staff burden and older adults' experiences, thereby constructing a multi-dimensional and long-term evaluation system for the effectiveness of nursing-technology collaboration; and establishing unified data interface standards, refining rules for responsibility allocation and ethical guidelines, and promoting the standardized integration of technology-generated data with the long-term care insurance system. By addressing these issues and actively drawing on mature international experience, it is expected that the sustainable development of nursing-technology collaboration in home-based rehabilitation can be ensured, with steady progress toward the ecological integration stage.

## References

[1] Chen M Li M Wan D Zha J. Global, regional, and national burden of depressive disorders in older adults from 1990 to 2021, with projections of prevalence to 2050: a trend analysis for the global burden of disease study 2021. J Affect Disord. (2026) 405:121531. 10.1016/j.jad.2026.121531 41780694

[2] Zhu X Wang Z Yang X Ning Z. About half of older adults have two or more chronic conditions at the same time: a systematic review and meta-analysis. Front Public Health. (2025) 13:1680745. 10.3389/fpubh.2025.1680745 41458407 PMC12738304

[3] Pulkki JM Aaltonen MS Jylhä MK Enroth LM. Is home always the best place to live? Preferences for place of residence and long-term care among the community-dwelling oldest old in Finland. Eur J Public Health. (2025) 35(6):1198–203. 10.1093/eurpub/ckaf196 41401366 PMC12707495

[4] Yun S Kwon S. Comparing health outcomes and costs of home and community-based services (HCBS) versus institutional care (IC) for long-term care insurance beneficiaries in South Korea: can the less severe benefit more? Health Policy. (2025) 161:105427. 10.1016/j.healthpol.2025.105427 40907060

[5] Elsheikh A Othman A Al-Thani D. Systematic analysis of cutting-edge technology for the wellbeing and safety of older persons. Disabil Rehabil Assist Technol. (2025) 20(8):2691–724. 10.1080/17483107.2025.2559185 41045132

[6] Lee J Lee H Choi M Kim JA. Care robots for community-dwelling older adults: an integrative review. Healthc Inform Res. (2025) 31(4):347–66. Conflict of Interest Mona Choi and Jung A Kim are members of the Editorial Board of Healthcare Informatics Research. They did not participate in the review or editorial decision for this manuscript. All other authors report no conflicts of interest. 10.4258/hir.2025.31.4.347 41265421 PMC12640725

[7] Salma I Testa D Mpoy JV Perez-Torrents J Rehault JM Cabanes E. Remote monitoring system for older adults at risk for complications: a scoping review. BMC Geriatr. (2025) 25(1):766. Declarations. Ethics approval and consent to participate: Not applicable. Consent for publication: Not applicable. Competing interests: The authors declare no competing interests. 10.1186/s12877-025-06385-8 41068645 PMC12512857

[8] Fu R Ma X. Understanding older adults’ adoption of smart product-service systems for medication management in China: an extended UTAUT model approach. Front Public Health. (2025) 13:1657473. 10.3389/fpubh.2025.1657473 41112639 PMC12532044

[9] Ohneberg C Stöbich N Warmbein A Rathgeber I Mehler-Klamt AC Fischer U. Assistive robotic systems in nursing care: a scoping review. BMC Nurs. (2023) 22(1):72. 10.1186/s12912-023-01230-y 36934280 PMC10024291

[10] Rantz MJ Marek KD Aud M Tyrer HW Skubic M Demiris G. A technology and nursing collaboration to help older adults age in place. Nurs Outlook. (2005) 53(1):40–5. 10.1016/j.outlook.2004.05.004 15761399

[11] Yong L Yue W Fuqiang L Baiqing S Kairu L. A multi-task allocation framework in elderly care scenarios with caregiver-robot collaboration. J Zhejiang Univ. (2025) 59(02):375–83.

[12] Walzer S Armbruster C Mahler S Farin-Glattacker E Kunze C. Factors influencing the implementation and adoption of digital nursing technologies: systematic umbrella review. J Med Internet Res. (2025) 27:e64616. 10.2196/64616 40743516 PMC12355146

[13] Rogers EM Singhal A Quinlan MM. Diffusion of innovations. In: Stacks DW Salwen MB, editors. An Integrated approach to Communication theory and Research. New York, NY: Routledge (2014). p. 432–48.

[14] Alexander GL Powell K Deroche CB Popejoy L Mosa ASM Koopman R. Building consensus toward a national nursing home information technology maturity model. J Am Med Inform Assoc. (2019) 26(6):495–505. 10.1093/jamia/ocz006 30889245 PMC7647342

[15] Alami H Lehoux P Shaw SE Papoutsi C Rybczynska-Bunt S Fortin J-P. Virtual care and the inverse care law: implications for policy, practice, research, public and patients. Int J Environ Res Public Health. (2022) 19(17):10591. 10.3390/ijerph191710591 36078313 PMC9518297

[16] Lee S-H Chon Y Kim Y-Y. Comparative analysis of long-term care in OECD countries: focusing on long-term care financing type. Healthcare. (2023) 11(2):206. The authors declare no conflict of interest. 10.3390/healthcare11020206 36673574 PMC9858923

[17] Murray CJ Frenk J. Organization WHO. A WHO framework for health system performance assessment. (1999).

[18] Shuchun L Xiuzhu Z Qian W Can C Kun Z. Thoughts on the development of designated long-term care insurance agencies——based on a survey of designated care institutions. Chin Health Insur. (2024) 2:5–10.

[19] Chong M. Healthy Tianjin long-term care insurance protection system. (2025):004.42422311

[20] Peters MDJ Marnie C Tricco AC Pollock D Munn Z Alexander L. Updated methodological guidance for the conduct of scoping reviews. JBI Evid Synth. (2020) 18(10):2119–26. 10.11124/JBIES-20-00167 33038124

[21] Tricco AC Lillie E Zarin W O'Brien KK Colquhoun H Levac D. PRISMA extension for scoping reviews (PRISMA-ScR): checklist and explanation. Ann Intern Med. (2018) 169(7):467–73. 10.7326/M18-0850 30178033

[22] Al Wachami N Chahboune M Youlyouz-Marfak I Mesradi MR Lemriss H Hilali A. Improving the quality of care and patient safety in oncology, the contribution of simulation-based training: a scoping review. Int J Nurs Sci. (2024) 11(2):187–96. 10.1016/j.ijnss.2024.03.005 38707685 PMC11064576

[23] Lockwood C Munn Z Porritt K. Qualitative research synthesis: methodological guidance for systematic reviewers utilizing meta-aggregation. JBI Evid Implement. (2015) 13(3):179–87. 10.1097/XEB.0000000000000062 26262565

[24] Keunjoo Y Jinhee S Eunhee C Seokwon H. The effects of ICT enhanced home-visit nursing in long-term care insurance on health-related quality of life among community-dwelling older adults. J Korean Acad Community Health Nurs. (2022) 33(1):1–12. 10.12799/jkachn.2022.33.1.1

[25] Jung HW Kim Y Kim H Kim M-k Lee H Park JY. Socially assistive robot hyodol for depressive symptoms of community-dwelling older adults in medically underserved areas: a preliminary study. J Clin Med. (2026) 15(1):217. 10.3390/jcm15010217 PMC12786537 41517468

[26] Zhao IY Leung AYM Huang Y Liu Y. A social robot in home care: acceptability and utility among community-dwelling older adults. Innov Aging. (2025) 9(5):igaf019. 10.1093/geroni/igaf019 40386025 PMC12082092

[27] Yixuan Z Min L Yan L. Multi-stakeholder collaboration in the long-term care service system: an international review from the perspective of digital technology. Chin Med Insur. (2025) 08:23–30. 10.19546/j.issn.1674-3830.2025.8.003

[28] Broadbent E Garrett J Jepsen N Li Ogilvie V Ahn HS Robinson H. Using robots at home to support patients with chronic obstructive pulmonary disease: pilot randomized controlled trial. J Med Internet Res. (2018) 20(2):e45. Conflicts of Interest: EB, BM, HSA, NK, and KP received funding from Yujin Robot Ltd for unrelated projects on transportation and aged care robots. The remaining authors declare that they have no competing interests. 10.2196/jmir.8640 29439942 PMC5829456

[29] Kiuchi K Otsu K Hayashi Y. Psychological insights into the research and practice of embodied conversational agents, chatbots and social assistive robots: a systematic meta-review. Behav Inf Technol. (2024) 43(15):3696–736. 10.1080/0144929X.2023.2286528

[30] Liedo B. Navigating autonomy, privacy, and ageism in robot home care with aged users: a preliminary analysis of ROB-IN. Bioethics. (2025) 39(5):448–55. 10.1111/bioe.13340 39081234 PMC12053780

[31] Bubeck M Keusgen CV Haltaufderheide J Klingler C Ranisch R. An interview study on socially assistive robots and professional care relationships. Nurs Ethics. (2026) 33:413–27. 10.1177/09697330251385025 41891306 PMC13009235

[32] Milella F Bandini S. Fostering artificial intelligence-based supports for informal caregivers: a systematic review of the literature. Intell Artif. (2024) 18(1):67–87. 10.3233/IA-240028

[33] Cingolani M Scendoni R Fedeli P Cembrani F. Artificial intelligence and digital medicine for integrated home care services in Italy: opportunities and limits. Front Public Health. (2023) 10:1095001. The authors declare that the research was conducted in the absence of any commercial or financial relationships that could be construed as a potential conflict of interest. 10.3389/fpubh.2022.1095001 36684935 PMC9849776

[34] Macdonald M Gallant A Weeks L Delahunty-Pike A Moody E Iduye D. Long-term care home residents’ experiences with socially assistive technologies and the effectiveness of these technologies: a mixed methods systematic review. JBI Evid Synth. (2024) 22(8):1410–59. 10.11124/JBIES-23-00021 38910530

[35] Donelle L Hiebert B Warner G Reid M Reid J Shariff S. Passive remote monitoring technologies’ influence on home care clients’ ability to stay home: multiprovincial randomized controlled trial. JMIR Aging. (2025) 8:e69107. Conflicts of Interest: None declared. 10.2196/69107 40106817 PMC11966072

[36] Koceska N Koceski S Beomonte Zobel P Trajkovik V Garcia N. A telemedicine robot system for assisted and independent living. Sensors. (2019) 19(4):834. The authors declare no conflict of interest. 10.3390/s19040834 30781647 PMC6412532

[37] Koceski S Koceska N. Evaluation of an assistive telepresence robot for elderly healthcare. J Med Syst. (2016) 40(5):121. 10.1007/s10916-016-0481-x 27037685

[38] Rantanen P Parkkari T Leikola S Airaksinen M Lyles A. An in-home advanced robotic system to manage elderly home-care patients’ medications: a pilot safety and usability study. Clin Ther. (2017) 39(5):1054–61. 10.1016/j.clinthera.2017.03.020 28433400

[39] Hongyan L. Research on the construction and application of the maturity model for digital transformation in vocational college education (Doctoral dissertation). Jiangxi Normal University (2025). CNKI. Available online at: https://link.cnki.net/doi/10.27178/d.cnki.gjxsu.2025.000038 (Accessed April 18, 2026).

[40] Vandemeulebroucke T Dierckx De Casterlé B Gastmans C. The use of care robots in aged care: a systematic review of argument-based ethics literature. Arch Gerontol Geriatr. (2018) 74:15–25. 10.1016/j.archger.2017.08.014 28926749

[41] Servaty R Kersten A Brukamp K Möhler R Mueller M. Implementation of robotic devices in nursing care. Barriers and facilitators: an integrative review. BMJ Open. (2020) 10(9):e038650. 10.1136/bmjopen-2020-038650 32958491 PMC7507851

[42] Shi W Zhou W Wang R Shen H Xu N Wang J. Exploring the perceptions and experiences of caregivers with the application of socially assistive robots in dementia care: a systematic review of qualitative studies. Int J Nurs Stud. (2025) 167:105084. 10.1016/j.ijnurstu.2025.105084 40262375

[43] Barrett JJ Jones CB. Task augmentation, automation, and hybridization in nursing: a conceptual framework for artificial intelligence-integrated care delivery. Nurs Outlook. (2025) 73(5):102524. 10.1016/j.outlook.2025.102524 40780090

[44] Abdelmohsen SA Al-jabri MM. Artificial intelligence applications in healthcare: a systematic review of their impact on nursing practice and patient outcomes. J Nurs Scholarsh. (2025) 57(6):957–66. 10.1111/jnu.70040 40836587

[45] Giorgi I Minutolo A Tirotto F Hagen O Esposito M Gianni M. I am robot, your health adviser for older adults: do you trust my advice? Int J Soc Robot. (2025) 17(5):883–902. 10.1007/s12369-023-01019-8

[46] Sun Y Chen J Ji M Li X. Wearable technologies for health promotion and disease prevention in older adults: systematic scoping review and evidence map. J Med Internet Res. (2025) 27:e69077. 10.2196/69077 40553512 PMC12238792

[47] Cai YJ Yao L Masaki H Shi TY. Evaluation methods of internet of things in nursing care interventions for older adults: a scoping review protocol. BMJ Open. (2025) 15(10):e101839. 10.1136/bmjopen-2025-101839 41161840 PMC12574410

[48] Gorce P Jacquier-Bret J. Fall detection in elderly people: a systematic review of ambient assisted living and smart home-related technology performance. Sensors. (2025) 25(21):6540. 10.3390/s25216540 41228764 PMC12609574

[49] Yeon BK Hong N Lee DW Lee Y. Caregiver burden and utilization of social welfare services of elderly dementia patients in Korean long-term care insurance system. Alzheimers Dement. (2011) 7(4, Supplement):S631–2. 10.1016/j.jalz.2011.05.1806

[50] Nishida T Honda A Fukuda K Ide Y Honda S. Risk factors for older people utilizing care services in the long-term care insurance system. Arch Gerontol Geriatr Plus. (2026) 3(1):100235. 10.1016/j.aggp.2025.100235

[51] Umegaki H Yanagawa M Nonogaki Z Nakashima H Kuzuya M Endo H. Burden reduction of caregivers for users of care services provided by the public long-term care insurance system in Japan. Arch Gerontol Geriatr. (2014) 58(1):130–3. 10.1016/j.archger.2013.08.010 24035002

[52] Curioso-Vílchez IC Fanti JR Presumido-Braccialli LM. Nível de satisfação do uso do eye tracker em uma pessoa com esclerose lateral amiotrófica (ELA): um estudo de caso. In: Paschoarelli LC Medola FO, editors. Tecnologia Assistiva: Pesquisa e Conhecimento II. 1st ed. Bauru: Canal 6 Editora (2018). p. 85–92.

[53] Curioso-Vílchez IC Presumido-Braccialli LM. Universal design and physical disability: perceptions on the use of support designs for smartphone by adults with cerebral palsy. Siglo Cero. (2023) 54(2):73–91. 10.14201/scero202354230264

[54] Curioso-Vílchez IC Presumido-Braccialli LM. Associations between the use of information and communication technologies and motor, manual and communication classification systems in adults with physical disability. In: Barredo Ibáñez D Gallardo-Echenique E Siringoringo H Lagares sDiez N, editors. Communication and Applied Technologies: Proceedings of ICOMTA 2024. Intelligent Innovation, Systems and Technologies (Singapore: Springer) (2025). Vol. 427. p. 107–16. 10.1007/978-981-96-0426-5_10

[55] Hainan L. Full implementation of long-term care insurance: leading service supply upgrades with unified standards. (2026):008. 10.28427/n.cnki.njjsb.2026.00075542524386

[56] Zilin X Lixiong Y. Building China’s elderly long-term care service system from a mixed welfare economy perspective—a comparison based on the practices of Germany, Japan, and South Korea. Administr Manag Reform. (2022) (05):93–103.

[57] Küpper-Nybelen J Ihle P Deetjen W Schubert I. Persons requiring long-term care: recommendation and utilization of rehabilitative therapies. Z Gerontol Geriatr. (2006) 39(2):100–8. 10.1007/s00391-006-0369-3 16622631

[58] Yulin L Kexin P. Choice of payment models, level assessment, and dynamic adjustment for long-term care insurance benefits. Fin Theory Explor. (2025) 05:67–80. 10.16620/j.cnki.jrjy.2025.05.006

[59] Tongtong J. Research on the fragmentation and integration of China’s long-term care insurance system (Phd thesis). Shanxi University of Finance and Economics (2025). CNKI. Available online at: https://link.cnki.net/doi/10.27283/d.cnki.gsxcc.2025.001707 (Accessed April 18, 2026).

[60] Xiao W. The accelerated development of the long-term care worker system. Doctoral Dissertation, Shanxi University of Finance and Economics. (2025):009. 10.28460/n.cnki.njrsb.2025.00511942422312

[61] Zhaoting Z Ziye X. Risks and regulation of intelligent assistive technology empowering elderly care services. Soc Work Manag. (2025) 25(06):83–90.

[62] Fan Z. Tech-institution interaction: the realistic picture structural conflicts, and synergy paths of AI empowering elderly care services. Popul Soc. (2025) 41(06):104–15.

