# Supplementary material for: A scoping review of “nursing-technology collaboration” in home-based rehabilitation for the elderly under the long-term care insurance system
Source: Front Med (Lausanne). 2026 Sep 25;13:1906465. doi: 10.3389/fmed.2026.1906465 (PMC13650299; doi:10.3389/fmed.2026.1906465)
Supplement: Supplementary file 1 [file Table1.docx]

**Identification of studies via databases and registers**

Records removed *before screening*:

Duplicate records removed (n =21)

Records marked as ineligible by automation tools (n =0 )

Records removed for other reasons (n =0 )

Records identified from:

Databases (n =146 )

PubMed(n=95)，Embase(n=18),

Cochrane Library (n=4),

Web of Science(n=6)，

ScienceDirect（n=11),’

Cinahl (n=12)

Cinahl（n=23)

**Identification**

Records screened

(n =125 )

Records excluded

(n =31 )

Reports sought for retrieval

(n = 94)

Reports not retrieved

(n = 0)

**Screening**

Reports excluded:

Research content does not align(n=66)、

Full text unavailable（n=13)

Reports assessed for eligibility

(n =94 )

Studies included in review:

RCT	(n =2 )

Quasi-experiment (n =5 )

Mixed methods (n = 1)

Qualitative Research( n=1)

**Systematic review / Scoping review / Meta-review** (n = 4)

**Theoretical / Policy analysis** (n = 2)

Studies included in review

(n =15 )

**Included**

Source: Page MJ, et al. BMJ 2021;372:n71. doi: 10.1136/bmj.n71.

This work is licensed under CC BY 4.0. To view a copy of this license, visit <https://creativecommons.org/licenses/by/4.0/>
