# Supplementary material for: A scoping review of “nursing-technology collaboration” in home-based rehabilitation for the elderly under the long-term care insurance system
Source: Front Med (Lausanne). 2026 Sep 25;13:1906465. doi: 10.3389/fmed.2026.1906465 (PMC13650299; doi:10.3389/fmed.2026.1906465)
Supplement: Supplementary file 2 [file Table2.docx]

Pubmed:

**((((((((((((((((((((((((Human-AI Collaboration) OR (Mobile Collaborative Nursing Robots)) OR (Technology-Assisted Nursing)) OR (Nurse-led innovations)) OR (Nursing-Technology Collaboration)) OR (Nurse-Technology Collaboration)) OR (Human-Machine Collaboration)) OR (Human-Robot Collaboration)) OR (Technology-Supported Care)) OR (Nurse-Digital Health Integration)) OR (Nursing Robots)) OR (Assistive Robots)) OR (Social Robots)) OR (Mobile Collaborative Nursing Robots)) OR (Human-AI Collaboration)) OR (Human-Machine Collaboration)) OR (Human-Robot Collaboration)) OR (Human-Robot Cohabitation)) OR (Nurse-Engineer Collaboration)) OR (Nurse-led Innovations)) OR (Technology-Assisted Nursing)) OR (Technology-Supported Care)) OR (Nurse-Digital Health Integration)) AND ((((((((((((((((((((Aged[MeSH Terms]) OR (Elderly[MeSH Terms])) OR (Frail Elderly[MeSH Terms])) OR (Elderly, Frail[MeSH Terms])) OR (Frail Elders[MeSH Terms])) OR (Elder, Frail[MeSH Terms])) OR (Elders, Frail[MeSH Terms])) OR (Frail Elder[MeSH Terms])) OR (Frail Older Adults[MeSH Terms])) OR (Adult, Frail Older[MeSH Terms])) OR (Adults, Frail Older[MeSH Terms])) OR (Frail Older Adult[MeSH Terms])) OR (Older Adult, Frail[MeSH Terms])) OR (Older Adults, Frail[MeSH Terms])) OR (Functionally-Impaired Elderly[MeSH Terms])) OR (Elderly, Functionally-Impaired[MeSH Terms])) OR (Functionally Impaired Elderly[MeSH Terms])) OR (Disabled Elderly[Title/Abstract])) OR (Impaired Elderly[Title/Abstract])) OR (Geriatric Patients[Title/Abstract]))) AND ((((((((((((((((((((((((((((((((((((((((((((((((((((((((((((((((((((((((((((((((((Home-Based Rehabilitation[Title/Abstract]) OR (Home Rehabilitation[Title/Abstract])) OR (Community Rehabilitation[Title/Abstract])) OR (Home Care Services[MeSH Terms])) OR (Home Care Service[MeSH Terms])) OR (Service, Home Care[MeSH Terms])) OR (Domiciliary Care[MeSH Terms])) OR (Care, Domiciliary[MeSH Terms])) OR (Services, Home Care[MeSH Terms])) OR (Care Services, Home[MeSH Terms])) OR (Home Care[MeSH Terms])) OR (Care, Home[MeSH Terms])) OR (Home Health Care[MeSH Terms])) OR (Home Care Services, Hospital-Based[MeSH Terms])) OR (Care, Hospital-Based Home[MeSH Terms])) OR (Care, Hospital Based Home[MeSH Terms])) OR (Hospital-Based Home Cares[MeSH Terms])) OR (Cares, Hospital-Based Home[MeSH Terms])) OR (Cares, Hospital Based Home[MeSH Terms])) OR (Home Care Services, Hospital Based[MeSH Terms])) OR (Home Care, Hospital-Based[MeSH Terms])) OR (Home Care, Hospital Based[MeSH Terms])) OR (Home Cares, Hospital-Based[MeSH Terms])) OR (Home Cares, Hospital Based[MeSH Terms])) OR (Hospital Based Home Cares[MeSH Terms])) OR (Hospital Home Care Services[MeSH Terms])) OR (Hospital-Based Home Care[MeSH Terms])) OR (Hospital Based Home Care[MeSH Terms])) OR (Hospital-Based Home Care Services[MeSH Terms])) OR (Hospital Based Home Care Services[MeSH Terms])) OR (Hospital in the Home[MeSH Terms])) OR (Hospital at Home[MeSH Terms])) OR (Home Care Agencies[MeSH Terms])) OR (Agencies, Home Care[MeSH Terms])) OR (Agency, Home Care[MeSH Terms])) OR (Care Agencies, Home[MeSH Terms])) OR (Care Agency, Home[MeSH Terms])) OR (Home Care Agency[MeSH Terms])) OR (Home Health Agencies[MeSH Terms])) OR (Agencies, Home Health[MeSH Terms])) OR (Agency, Home Health[MeSH Terms])) OR (Home Health Agency[MeSH Terms])) OR (Home Health Care Agencies[MeSH Terms])) OR (Foster Home Care[MeSH Terms])) OR (Care, Foster Home[MeSH Terms])) OR (Fostering[MeSH Terms])) OR (Adult Foster Care[MeSH Terms])) OR (Care, Adult Foster[MeSH Terms])) OR (Foster Care, Adult[MeSH Terms])) OR (Kinship Care[MeSH Terms])) OR (Care, Kinship[MeSH Terms])) OR (Home Health Aides[MeSH Terms])) OR (Aide, Home Health[MeSH Terms])) OR (Aides, Home Health[MeSH Terms])) OR (Health Aide, Home[MeSH Terms])) OR (Health Aides, Home[MeSH Terms])) OR (Home Health Aide[MeSH Terms])) OR (Home Care Aides[MeSH Terms])) OR (Aide, Home Care[MeSH Terms])) OR (Aides, Home Care[MeSH Terms])) OR (Care Aide, Home[MeSH Terms])) OR (Care Aides, Home[MeSH Terms])) OR (Home Care Aide[MeSH Terms])) OR (Homemaker-Home Health Aides[MeSH Terms])) OR (Aide, Homemaker-Home Health[MeSH Terms])) OR (Aides, Homemaker-Home Health[MeSH Terms])) OR (Health Aide, Homemaker-Home[MeSH Terms])) OR (Health Aides, Homemaker-Home[MeSH Terms])) OR (Homemaker-Home Health Aide[MeSH Terms])) OR (Homemaker Home Health Aides[MeSH Terms])) OR (Home Nursing[MeSH Terms])) OR (Home Care, Non-Professional[MeSH Terms])) OR (Care, Non-Professional Home[MeSH Terms])) OR (Home Care, Non Professional[MeSH Terms])) OR (Non-Professional Home Care[MeSH Terms])) OR (Nursing, Home[MeSH Terms])) OR (Home Care, Nonprofessional[MeSH Terms])) OR (Care, Nonprofessional Home[MeSH Terms])) OR (Nonprofessional Home Care[MeSH Terms])) OR (Home Health Nursing[MeSH Terms])) OR (Nursing, Home Health[MeSH Terms])) OR (Home Health Care Nursing[MeSH Terms]))**

**Cochrane library:**

**#1 MeSH descriptor: [Insurance, Long-Term Care] explode all trees**

**#2 ("Long Term Care Insurance" OR "Insurance" OR "Long Term Care" OR "Long-Term Care Insurance" OR "Insurance, Long-Term Care"):ti,ab,kw**

**#3 #1 OR #2**

**#4 MeSH descriptor: [Home Care Services] explode all trees**

**#5 ("Home Care Services" OR "Care Services, Home" OR "Home Care Service" OR "Care, Domiciliary" OR "Domiciliary Care" OR "Service, Home Care" OR "Services, Home Care" OR "Home Health Care" OR "Home Care" OR "Care, Home"):ti,ab,kw**

**#6 MeSH descriptor: [Home Care Services, Hospital-Based] explode all trees**

**#7 (("Home Care Services, Hospital-Based" OR "Hospital-Based Home Cares" OR "Hospital Based Home Cares" OR "Hospital at Home" OR "Hospital in the Home" OR "Hospital Based Home Care Services" OR "Hospital-Based Home Care" OR "Hospital-Based Home Care Services" OR "Care, Hospital Based Home" OR "Home Cares, Hospital-Based" OR "Hospital Home Care Services" OR "Home Care, Hospital Based" OR "Hospital Based Home Care" OR "Home Cares, Hospital Based" OR "Care, Hospital-Based Home" OR "Cares, Hospital Based Home" OR "Home Care Services, Hospital Based" OR "Cares, Hospital-Based Home" OR "Home Care, Hospital-Based")):ti,ab,kw**

**#8 MeSH descriptor: [Home Care Agencies] explode all trees**

**#9 (“Home Care Agencies” OR "Agencies, Home Care" OR "Care Agency, Home" OR "Home Care Agency" OR "Care Agencies, Home" OR "Agency, Home Care" OR "Home Health Care Agencies" OR "Agency, Home Health" OR "Home Health Agencies" OR "Agencies, Home Health" OR "Home Health Agency"):ti,ab,kw**

**#10 MeSH descriptor: [Foster Home Care] explode all trees**

**#11 ("Foster Home Care" OR "Adult Foster Care" OR "Foster Care, Adult; Care, Adult Foster" OR "Care, Foster Home" OR "Care, Kinship" OR "Kinship Care" OR "Fostering"):ti,ab,kw**

**#12 MeSH descriptor: [Home Nursing] in all MeSH products**

**#13 ("Home Nursing" OR "Nonprofessional Home Care" OR "Non-Professional Home Care" OR "Nursing, Home" OR "Care, Non-Professional Home" OR "Home Care, Non Professional" OR "Home Care, Nonprofessional" OR "Care, Nonprofessional Home" OR "Home Care, Non-Professional" OR "Nurses, Community Health" OR " Health Visitor" OR "Nurses, Home Health" OR "Nurse, Home" OR "Home Nurse" OR "Nurses, Home" OR "Home Health Nurse" OR "Visiting Nurses" OR "Home Health Nurses" OR "Nurse, Home Health" OR "Visiting Nurse" OR "Nurse, Visiting" OR "Home Nurses" OR "Nurses, Visiting" OR "Nurse, Community Health" OR "Community Health Nurse" OR "Community Health Nurses"):ti,ab,kw**

**#14 MeSH descriptor: [Home Health Nursing] in all MeSH products**

**#15 ("Home Health Nursing" OR "Home Health Care Nursing" OR "Nursing, Home Health" OR "Nurses, Community Health" OR "Health Visitor" OR " Health Visitors" OR "Nurses, Home Health" OR "Nurse, Home" OR "Home Nurse" OR "Nurses, Home" OR "Home Health Nurse" OR "Visiting Nurses" OR "Home Health Nurses" OR "Nurse, Home Health" OR "Visiting Nurse" OR "Nurse, Visiting" OR "Home Nurses" OR "Nurses, Visiting" OR "Nurse, Community Health" OR "Community Health Nurse" OR "Community Health Nurses"):ti,ab,kw**

**#16 #4 OR #5 OR #6 OR #7 OR #8 OR #9 OR #10 OR #11 OR #12 OR #13 OR #14 OR #15**

**#17 MeSH descriptor: [Aged] in all MeSH products**

**#18 ("Aged" OR " Elderly" OR "Aging" OR "Senescence" OR "Biological Aging" OR "Aging, Biological"):ti,ab,kw**

**#19 MeSH descriptor: [Frail Elderly] explode all trees**

**#20 ("Frail Elderly" OR "Functionally Impaired Elderly" OR "Elderly, Functionally-Impaired" OR "Functionally-Impaired Elderly" OR "Older Adult, Frail" OR "Frail Older Adults" OR "Adults, Frail Older" OR "Older Adults, Frail" OR "Frail Older Adult" OR " Adult, Frail Older" OR "Elders, Frail" OR "Frail Elder" OR "Elder, Frail" OR "Elderly, Frail" OR "Frail Elders"):ti,ab,kw**

**#21 #17 OR #18 OR #19 OR #20**

**#22 ("Human-AI Collaboration" OR "Mobile Collaborative Nursing Robots" OR "Technology-Assisted Nursing" OR "Nurse-led innovations" OR "Nursing-Technology Collaboration" OR "Nurse-Technology Collaboration" OR "Human-Machine Collaboration" OR "Human-Robot Collaboration" OR "Technology-Supported Care" OR "Nurse-Digital Health Integration" OR "Collaborative Care with Technology" OR "Nurse-Remote Monitoring Integration"):ti,ab,kw**

**#23 (“Nursing Robots” OR "Assistive Robots" OR "Social Robots" OR "Mobile Collaborative Nursing Robots" OR "Human-AI Collaboration" OR "Human-Machine Collaboration" OR "Human-Robot Collaboration" OR "Human-Robot Cohabitation" OR "Nurse-Engineer Collaboration" OR "Nurse-led Innovations" OR "Technology-Assisted Nursing" OR "Technology-Supported Care" OR "Nurse-Digital Health Integration"):ti,ab,kw**

**#24 #22 OR #23**

**#25 #3 AND #16 AND #21 AND #24**

WEB OF SCIENCE:

#1 **"Insurance, Long-Term Care"** (主题) or **"Long Term Care Insurance" OR "Insurance" OR "Long Term Care" OR "Long-Term Care Insurance" OR "Insurance, Long-Term Care"** (主题)

#2 **"Home Care Services"** (主题) or **"Home Care Services" OR "Care Services, Home" OR "Home Care Service" OR "Care, Domiciliary" OR "Domiciliary Care" OR "Service, Home Care" OR "Services, Home Care" OR "Home Health Care" OR "Home Care" OR "Care, Home"** (主题) or **"Home Care Services, Hospital-Based"** (主题) or **"Home Care Services, Hospital-Based" OR "Hospital-Based Home Cares" OR "Hospital Based Home Cares" OR "Hospital at Home" OR "Hospital in the Home" OR "Hospital Based Home Care Services" OR "Hospital-Based Home Care" OR "Hospital-Based Home Care Services" OR "Care, Hospital Based Home" OR "Home Cares, Hospital-Based" OR "Hospital Home Care Services" OR "Home Care, Hospital Based" OR "Hospital Based Home Care" OR "Home Cares, Hospital Based" OR "Care, Hospital-Based Home" OR "Cares, Hospital Based Home" OR "Home Care Services, Hospital Based" OR "Cares, Hospital-Based Home" OR "Home Care, Hospital-Based"** (主题) or **“Home Care Agencies” OR "Agencies, Home Care" OR "Care Agency, Home" OR "Home Care Agency" OR "Care Agencies, Home" OR "Agency, Home Care" OR "Home Health Care Agencies" OR "Agency, Home Health" OR "Home Health Agencies" OR "Agencies, Home Health" OR "Home Health Agency"** (主题) or **"Foster Home Care" OR "Adult Foster Care" OR "Foster Care, Adult; Care, Adult Foster" OR "Care, Foster Home" OR "Care, Kinship" OR "Kinship Care" OR "Fostering"** (主题) or **"Home Nursing" OR "Nonprofessional Home Care" OR "Non-Professional Home Care" OR "Nursing, Home" OR "Care, Non-Professional Home" OR "Home Care, Non Professional" OR "Home Care, Nonprofessional" OR "Care, Nonprofessional Home" OR "Home Care, Non-Professional" OR "Nurses, Community Health" OR " Health Visitor" OR "Nurses, Home Health" OR "Nurse, Home" OR "Home Nurse" OR "Nurses, Home" OR "Home Health Nurse" OR "Visiting Nurses" OR "Home Health Nurses" OR "Nurse, Home Health" OR "Visiting Nurse" OR "Nurse, Visiting" OR "Home Nurses" OR "Nurses, Visiting" OR "Nurse, Community Health" OR "Community Health Nurse" OR "Community Health Nurses"** (主题) or **"Home Health Nursing" OR "Home Health Care Nursing" OR "Nursing, Home Health" OR "Nurses, Community Health" OR "Health Visitor" OR " Health Visitors" OR "Nurses, Home Health" OR "Nurse, Home" OR "Home Nurse" OR "Nurses, Home" OR "Home Health Nurse" OR "Visiting Nurses" OR "Home Health Nurses" OR "Nurse, Home Health" OR "Visiting Nurse" OR "Nurse, Visiting" OR "Home Nurses" OR "Nurses, Visiting" OR "Nurse, Community Health" OR "Community Health Nurse" OR "Community Health Nurses"** (主题)

#3 **"Aged" OR " Elderly" OR "Aging" OR "Senescence" OR "Biological Aging" OR "Aging, Biological"** (主题) or **"Frail Elderly" OR "Functionally Impaired Elderly" OR "Elderly, Functionally-Impaired" OR "Functionally-Impaired Elderly" OR "Older Adult, Frail" OR "Frail Older Adults" OR "Adults, Frail Older" OR "Older Adults, Frail" OR "Frail Older Adult" OR " Adult, Frail Older" OR "Elders, Frail" OR "Frail Elder" OR "Elder, Frail" OR "Elderly, Frail" OR "Frail Elders"** (主题)

#4 **"Human-AI Collaboration" OR "Mobile Collaborative Nursing Robots" OR "Technology-Assisted Nursing" OR "Nurse-led innovations" OR "Nursing-Technology Collaboration" OR "Nurse-Technology Collaboration" OR "Human-Machine Collaboration" OR "Human-Robot Collaboration" OR "Technology-Supported Care" OR "Nurse-Digital Health Integration" OR "Collaborative Care with Technology" OR "Nurse-Remote Monitoring Integration"** (主题) or **“Nursing Robots” OR "Assistive Robots" OR "Social Robots" OR "Mobile Collaborative Nursing Robots" OR "Human-AI Collaboration" OR "Human-Machine Collaboration" OR "Human-Robot Collaboration" OR "Human-Robot Cohabitation" OR "Nurse-Engineer Collaboration" OR "Nurse-led Innovations" OR "Technology-Assisted Nursing" OR "Technology-Supported Care" OR "Nurse-Digital Health Integration"** (主题)

#5 #1 AND #2 AND #3 AND #4

SCIENCEDIRECT:

("long-term care" OR "long-term care insurance") AND ("home rehabilitation" OR "home care") AND ("aged" OR "older adults") AND ("human-AI collaboration" OR "nurse-technology collaboration")

Cinahl:

S4：**[AB ("Home Care Services" OR "Care Services, Home" OR "Home Care Service" OR "Care, Domiciliary" OR "Domiciliary Care" OR "Service, Home Care" OR "Services, Home Care" OR "Home Health Care" OR "Home Care" OR "Care, Home") OR AB ("Home Care Services, Hospital-Based" OR "Hospital-Based Home Cares" OR "Hospital Based Home Cares" OR "Hospital at Home" OR "Hospital in the Home" OR "Hospital Based Home Care Services" OR "Hospital-Based Home Care" OR "Hospital-Based Home Care Services" OR "Care, Hospital Based Home" OR "Home Cares, Hospital-Based" OR "Hospital Home Care Services" OR "Home Care, Hospital Based" OR "Hospital Based Home Care" OR "Home Cares, Hospital Based" OR "Care, Hospital-Based Home" OR "Cares, Hospital Based Home" OR "Home Care Services, Hospital Based" OR "Cares, Hospital-Based Home" OR "Home Care, Hospital-Based") OR AB (“Home Care Agencies” OR "Agencies, Home Care" OR "Care Agency, Home" OR "Home Care Agency" OR "Care Agencies, Home" OR "Agency, Home Care" OR "Home Health Care Agencies" OR "Agency, Home Health" OR "Home Health Agencies" OR "Agencies, Home Health" OR "Home Health Agency") OR AB ("Foster Home Care" OR "Adult Foster Care" OR "Foster Care, Adult; Care, Adult Foster" OR "Care, Foster Home" OR "Care, Kinship" OR "Kinship Care" OR "Fostering") OR AB ("Home Nursing" OR "Nonprofessional Home Care" OR "Non-Professional Home Care" OR "Nursing, Home" OR "Care, Non-Professional Home" OR "Home Care, Non Professional" OR "Home Care, Nonprofessional" OR "Care, Nonprofessional Home" OR "Home Care, Non-Professional" OR "Nurses, Community Health" OR " Health Visitor" OR "Nurses, Home Health" OR "Nurse, Home" OR "Home Nurse" OR "Nurses, Home" OR "Home Health Nurse" OR "Visiting Nurses" OR "Home Health Nurses" OR "Nurse, Home Health" OR "Visiting Nurse" OR "Nurse, Visiting" OR "Home Nurses" OR "Nurses, Visiting" OR "Nurse, Community Health" OR "Community Health Nurse" OR "Community Health Nurses") OR AB ("Home Health Nursing" OR "Home Health Care Nursing" OR "Nursing, Home Health" OR "Nurses, Community Health" OR "Health Visitor" OR " Health Visitors" OR "Nurses, Home Health" OR "Nurse, Home" OR "Home Nurse" OR "Nurses, Home" OR "Home Health Nurse" OR "Visiting Nurses" OR "Home Health Nurses" OR "Nurse, Home Health" OR "Visiting Nurse" OR "Nurse, Visiting" OR "Home Nurses" OR "Nurses, Visiting" OR "Nurse, Community Health" OR "Community Health Nurse" OR "Community Health Nurses")](https://research.ebsco.com/search/results?db=ccm,pdh,psyh,pbh,sbh,ddh,nlebk,ram,bwh,e600xww,bsh,e001mww,rjz,rph&expanders=concept&limiters=rjz.PT1:Y&searchMode=boolean&sort=relevance&sqId=sq:542df5b6-a7c6-4402-82d6-e49327bf64b1&userDirectAction=true)**

### **S5:[AB ("Aged" OR " Elderly" OR "Aging" OR "Senescence" OR "Biological Aging" OR "Aging, Biological") OR AB ("Frail Elderly" OR "Functionally Impaired Elderly" OR "Elderly, Functionally-Impaired" OR "Functionally-Impaired Elderly" OR "Older Adult, Frail" OR "Frail Older Adults" OR "Adults, Frail Older" OR "Older Adults, Frail" OR "Frail Older Adult" OR " Adult, Frail Older" OR "Elders, Frail" OR "Frail Elder" OR "Elder, Frail" OR "Elderly, Frail" OR "Frail Elders")](https://research.ebsco.com/search/results?db=ccm,pdh,psyh,pbh,sbh,ddh,nlebk,ram,bwh,e600xww,bsh,e001mww,rjz,rph&expanders=concept&limiters=rjz.PT1:Y&searchMode=boolean&sort=relevance&sqId=sq:b99f74eb-4c1a-4950-b7a0-ee5dc672502f&userDirectAction=true)**

### **S6:[AB ("Human-AI Collaboration" OR "Mobile Collaborative Nursing Robots" OR "Technology-Assisted Nursing" OR "Nurse-led innovations" OR "Nursing-Technology Collaboration" OR "Nurse-Technology Collaboration" OR "Human-Machine Collaboration" OR "Human-Robot Collaboration" OR "Technology-Supported Care" OR "Nurse-Digital Health Integration" OR "Collaborative Care with Technology" OR "Nurse-Remote Monitoring Integration") OR AB (“Nursing Robots” OR "Assistive Robots" OR "Social Robots" OR "Mobile Collaborative Nursing Robots" OR "Human-AI Collaboration" OR "Human-Machine Collaboration" OR "Human-Robot Collaboration" OR "Human-Robot Cohabitation" OR "Nurse-Engineer Collaboration" OR "Nurse-led Innovations" OR "Technology-Assisted Nursing" OR "Technology-Supported Care" OR "Nurse-Digital Health Integration")](https://research.ebsco.com/search/results?db=ccm,pdh,psyh,pbh,sbh,ddh,nlebk,ram,bwh,e600xww,bsh,e001mww,rjz,rph&expanders=concept&limiters=rjz.PT1:Y&searchMode=boolean&sort=relevance&sqId=sq:22610019-4722-4dcd-9f0b-77fccb2307e2&userDirectAction=true)**

### **[(((S6)) AND (S5)) AND (S4)](https://research.ebsco.com/search/results?combinedSearchQueryId=sq:6fa7796e-5225-4a65-9edd-2e08c1e308dc&db=ccm,pdh,psyh,pbh,sbh,ddh,nlebk,ram,bwh,e600xww,bsh,e001mww,rjz,rph&expanders=concept&limiters=rjz.PT1:Y&searchMode=boolean&sort=relevance&sqId=sq:6fa7796e-5225-4a65-9edd-2e08c1e308dc&userDirectAction=true)**

**EMBASE:**

foster care

'foster home' OR 'foster home care' OR 'foster care'

#1 **'frail elderly'**:ab,ti

#2 **'aged'**/exp

#3 **aged**:ab,ti OR **'aged patient'**:ab,ti OR **'aged people'**:ab,ti OR **'aged person'**:ab,ti OR **'aged subject'**:ab,ti OR **elderly**:ab,ti OR **'elderly patient'**:ab,ti OR **'elderly people'**:ab,ti OR **'elderly person'**:ab,ti OR **'elderly subject'**:ab,ti OR **'senior citizen'**:ab,ti OR **senium**:ab,ti

#5 #1 OR #2 OR #3

#6 **'home care'**/exp 19

#7 **'domestic health care'**:ab,ti OR **'domestic healthcare'**:ab,ti OR **'domiciliary care'**:ab,ti OR **'domiciliary health care'**:ab,ti OR **'domiciliary healthcare'**:ab,ti OR **'home care agencies'**:ab,ti OR **'home care program'**:ab,ti OR **'home care programme'**:ab,ti OR **'home care service'**:ab,ti OR **'home care services'**:ab,ti OR **'home care services, hospital-based'**:ab,ti OR **'home health care'**:ab,ti OR **'home health nursing'**:ab,ti OR **'home healthcare'**:ab,ti OR **'home help'**:ab,ti OR **'home nursing'**:ab,ti OR **'home service'**:ab,ti OR **'home treatment'**:ab,ti OR **homecare**:ab,ti OR **'homemaker service'**:ab,ti OR **'homemaker services'**:ab,ti OR **'hospital-based home care services'**:ab,ti OR **'home care'**:ab,ti 22

#8 **'foster care'**/exp	25

\# 9 **'foster home'**:ab,ti OR **'foster home care'**:ab,ti OR **'foster care'**:ab,ti 26

#10 #6 OR #7 OR #8 OR #9	#36

#11 **'nursing-technology collaboration'**:ab,ti OR **'nurse-technology collaboration'**:ab,ti OR **'collaborative care with technology'**:ab,ti OR **'nurse-remote monitoring integration'**:ab,ti OR **'nursing robots'**:ab,ti OR **'assistive robots'**:ab,ti OR **'social robots'**:ab,ti OR **'mobile collaborative nursing robots'**:ab,ti OR **'human-ai collaboration'**:ab,ti OR **'human-machine collaboration'**:ab,ti OR **'human-robot collaboration'**:ab,ti OR **'human-robot cohabitation'**:ab,ti OR **'nurse-engineer collaboration'**:ab,ti OR **'nurse-led innovations'**:ab,ti OR **'technology-assisted nursing'**:ab,ti OR **'technology-supported care'**:ab,ti OR **'nurse-digital health integration'**:ab,ti

#12 #5 AND #10 AND #1
